# Supplementary material for: Selenoxides as Excellent Chalcogen Bond Donors: Effect of Metal Coordination
Source: Molecules. 2022 Dec 13;27(24):8837. doi: 10.3390/molecules27248837 (PMC9785337; doi:10.3390/molecules27248837)
Supplement: Supplementary file 1 [file molecules-27-08837-s001.zip › molecules-2090054-supplementary.pdf]

Supplementary Material for manuscript

Selenoxides as excellent chalcogen bond donors: Effect of met-al coordination

Sergi Burguera[1], Rosa M. Gomila[1], Antonio Bauzá[1] and Antonio Frontera[1,*]

[1] Universitat de les Illes Balears, Crta de Valldemossa km 7.5, 0722 Palma de Mallorca (Baleares), SPAIN
* Correspondence: toni.frontera@uib.es

Figure S1

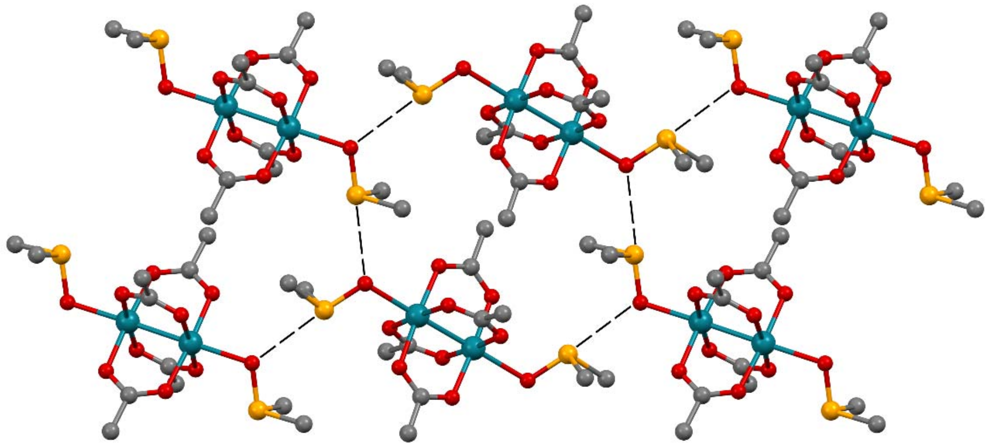

Figure S1. 2D assembly directed by ChBs in the solid state of RUZCIY. ChBs represented as dashed lines

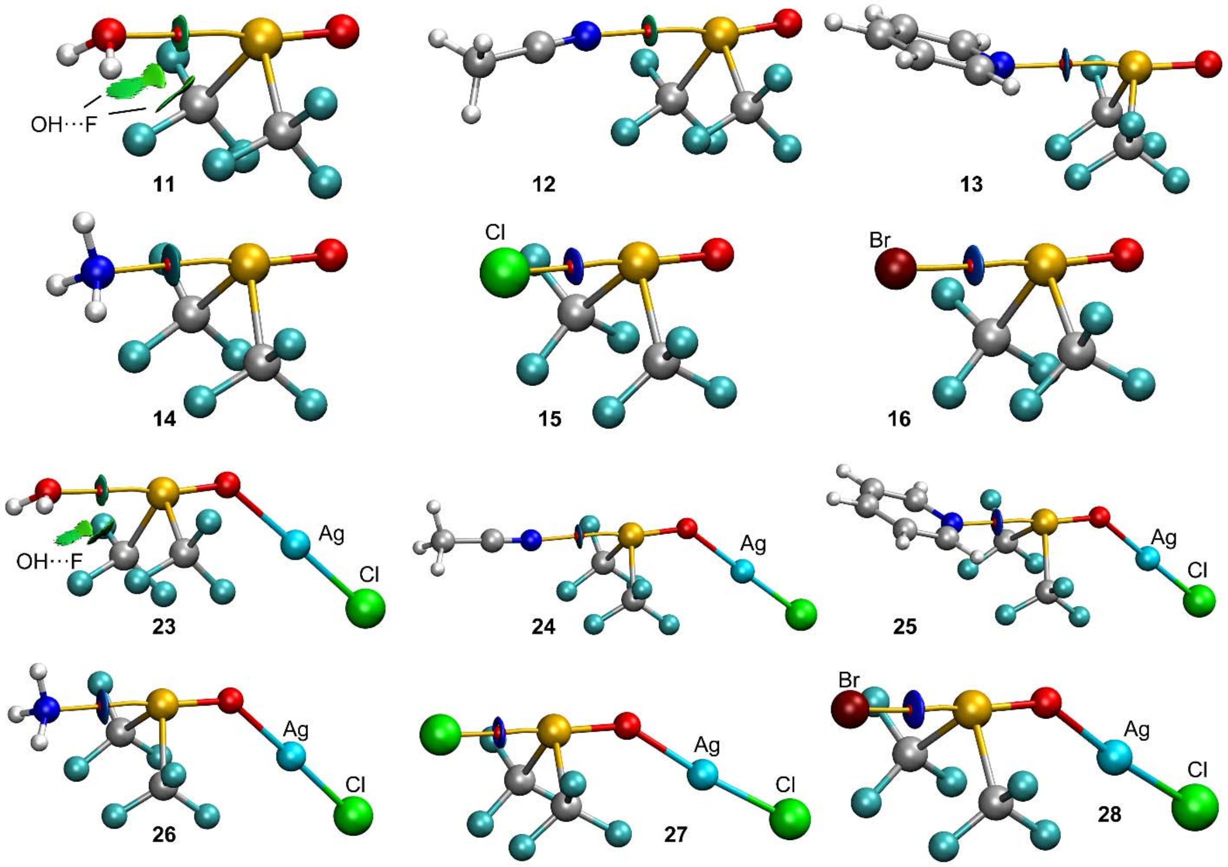

Figure S2. Combined QTAIM (bond critical points as red spheres and bond paths as yellow lines) and NCIPlot analyses of complexes **1–16** and **23–28** at the PBE0-D3/def2-TZVP level of theory. Only intermolecular interactions are represented.

Cartesian Coordinates

```
1
Se     0.7222617   -0.8955764    0.0000000
O     -0.3014888   -2.1758406    0.0000000
C      0.0403377    0.2442997    1.4272748
C      0.0403377    0.2442997   -1.4272748
H      0.2769065   -0.2471246    2.3696465
H      0.5120022    1.2265329    1.3808012
H     -1.0396329    0.3120004    1.2988705
H     -1.0396329    0.3120004   -1.2988705
H      0.5120022    1.2265329   -1.3808012
H      0.2769065   -0.2471246   -2.3696465

2
Se     0.2807152   -1.2184856    0.0000000
O     -1.1700801   -1.9402714    0.0000000
C      0.1263906    0.1817779    1.4821167
C      0.1263906    0.1817779   -1.4821167
F      0.1757815   -0.4802112    2.6304326
F      1.1484792    1.0274570    1.4276350
F     -1.0059688    0.8503548    1.4185034
F     -1.0059688    0.8503548   -1.4185034
F      1.1484792    1.0274570   -1.4276350
F      0.1757815   -0.4802112   -2.6304326
3
Se     1.1271473   -0.8955929    0.0000000
O     -0.2001046   -1.9075233    0.0000000
C      0.7397810    0.3422488    1.4354205
H     -0.2802003    0.7044965    1.2939413
H      1.4607926    1.1600487    1.4094115
H      0.8243382   -0.2122054    2.3685549
```

C	0.7397810	0.3422488	-1.4354205
H	0.8243382	-0.2122054	-2.3685549
H	1.4607926	1.1600487	-1.4094115
H	-0.2802003	0.7044965	-1.2939413
Ag	-2.2063155	-1.1341613	0.0000000
Cl	-4.2101502	-0.0518997	0.0000000

4

Se	0.8961528	-1.1742389	0.0000000
O	-0.6323545	-1.7761967	0.0000000
C	0.8555054	0.2122517	1.5026716
C	0.8555054	0.2122517	-1.5026716
Ag	-2.5795369	-0.7527470	0.0000000
Cl	-4.5530317	0.3819923	0.0000000
F	0.8350342	-0.4741697	2.6303016
F	1.9523388	0.9476782	1.4471864
F	-0.2084932	0.9748349	1.4276567
F	-0.2084932	0.9748349	-1.4276567
F	1.9523388	0.9476782	-1.4471864
F	0.8350342	-0.4741697	-2.6303016

5

Se	-1.0221653	-0.7199958	0.0000000
O	-2.6602695	-0.6162667	0.0000000
C	-0.4768842	0.4944476	1.4227194
H	-1.0598472	1.4069626	1.3007753
H	0.5935434	0.6819416	1.3442931
H	-0.7238734	0.0171345	2.3698923
C	-0.4768842	0.4944476	-1.4227194
H	-0.7238734	0.0171345	-2.3698923
H	0.5935434	0.6819416	-1.3442931
H	-1.0598472	1.4069626	-1.3007753
O	2.2754655	-0.9029218	0.0000000
H	2.3705460	-1.4808941	-0.7610148
H	2.3705460	-1.4808941	0.7610148

6

Se	-1.7315861	-0.8468522	0.0000000
O	-3.2559706	-1.4594919	0.0000000
C	-1.7595729	0.4781078	1.4263394
H	-2.6609788	1.0753875	1.2924341
H	-0.8548423	1.0826155	1.3706716
H	-1.8064687	-0.0670738	2.3679318
C	-1.7595729	0.4781078	-1.4263394
H	-1.8064687	-0.0670738	-2.3679318
H	-0.8548423	1.0826155	-1.3706716
H	-2.6609788	1.0753875	-1.2924341
N	1.4211099	0.6744236	0.0000000
C	2.3862426	0.0515212	0.0000000
C	3.6014544	-0.7366932	0.0000000
H	3.6348026	-1.3702675	0.8875430
H	3.6348026	-1.3702675	-0.8875430
H	4.4728701	-0.0804463	0.0000000

7

Se	-2.4990537	-0.7366620	0.0000000
O	-4.0994221	-1.1063835	0.0000000
C	-2.3372635	0.5877260	1.4211313
H	-3.1682445	1.2822468	1.3003618
H	-1.3732149	1.0872515	1.3356692
H	-2.4258965	0.0600071	2.3695776
C	-2.3372635	0.5877260	-1.4211313
H	-2.4258965	0.0600071	-2.3695776
H	-1.3732149	1.0872515	-1.3356692
H	-3.1682445	1.2822468	-1.3003618
N	0.7512261	0.0353545	0.0000000
C	1.4161920	-0.1459677	1.1363483
C	1.4161920	-0.1459677	-1.1363483
C	2.7547172	-0.5044832	1.1913866
H	0.8505282	0.0006918	2.0527539
C	2.7547172	-0.5044832	-1.1913866
H	0.8505282	0.0006918	-2.0527539
C	3.4384775	-0.6855916	0.0000000
H	3.2447327	-0.6380808	2.1481214
H	3.2447327	-0.6380808	-2.1481214
H	4.4856710	-0.9655003	0.0000000

8

Se	-1.2111587	-0.7936295	0.0000000
O	-2.8519753	-0.8512562	0.0000000
C	-0.7806569	0.4617744	1.4238969
H	-1.4173688	1.3355390	1.2881706
H	0.2781085	0.7102962	1.3587971
H	-1.0129777	-0.0293192	2.3676900
C	-0.7806569	0.4617744	-1.4238969
H	-1.0129777	-0.0293192	-2.3676900
H	0.2781085	0.7102962	-1.3587971
H	-1.4173688	1.3355390	-1.2881706
N	2.2613350	-0.6494409	0.0000000
H	1.9642192	-1.6181026	0.0000000
H	2.8516848	-0.5220758	0.8133203
H	2.8516848	-0.5220758	-0.8133203

9

Se	-0.6584276	-1.0492202	0.0000000
O	-2.3106429	-0.9132879	0.0000000
C	-0.0763196	0.1352758	1.4207393
H	-0.6303545	1.0675352	1.3038234
H	1.0077388	0.2561728	1.2988622
H	-0.3324300	-0.3444748	2.3651683
C	-0.0763196	0.1352758	-1.4207393
H	-0.3324300	-0.3444748	-2.3651683
H	1.0077388	0.2561728	-1.2988622
H	-0.6303545	1.0675352	-1.3038234
Cl	3.0318011	-0.2665099	0.0000000

10

Se	-0.6636046	-1.0613900	0.0000000
O	-2.3156048	-0.9430665	0.0000000
C	-0.0996411	0.1291892	1.4242834
H	-0.6788617	1.0473059	1.3195034
H	0.9787238	0.2863462	1.3087322
H	-0.3345541	-0.3645262	2.3668851
C	-0.0996411	0.1291892	-1.4242834
H	-0.3345541	-0.3645262	-2.3668851
H	0.9787238	0.2863462	-1.3087322
H	-0.6788617	1.0473059	-1.3195034
Br	3.2478755	-0.1921737	0.0000000

11

Se	-1.0574070	-0.8059399	0.0000000
O	-2.6614217	-0.5691682	0.0000000
C	-0.4556707	0.4751237	1.4869311
C	-0.4556707	0.4751237	-1.4869311
O	1.9166401	-1.5819103	0.0000000
H	2.3710843	-1.2122366	-0.7613558
H	2.3710843	-1.2122366	0.7613558
F	-0.6726540	-0.1570675	-2.6359063
F	-1.1755466	1.5778395	1.4617330
F	-1.1755466	1.5778395	-1.4617330
F	0.8338813	0.7948501	-1.4210859
F	0.8338813	0.7948501	1.4210859
F	-0.6726540	-0.1570675	2.6359063

12

Se	-1.9629810	-1.0351563	0.0000000
O	-3.5569627	-1.3387521	0.0000000
C	-1.8032958	0.3686563	1.4854465
C	-1.8032958	0.3686563	-1.4854465
N	1.2542436	-0.8857917	0.0000000
C	2.3691601	-0.6120186	0.0000000
C	3.7748842	-0.2636565	0.0000000
H	4.2603442	-0.6712897	0.8878731
H	4.2603442	-0.6712897	-0.8878731
H	3.8894482	0.8212693	0.0000000
F	-1.7997787	-0.3028004	2.6335626
F	-0.6932995	1.0875236	1.4099529
F	-2.8478664	1.1749631	1.4671746
F	-2.8478664	1.1749631	-1.4671746
F	-0.6932995	1.0875236	-1.4099529
F	-1.7997787	-0.3028004	-2.6335626

13

Se	-2.2516281	-0.9707095	0.0000000

O	-3.7980486	-1.4630520	0.0000000
C	-2.2886868	0.4313688	1.5045432
C	-2.2886868	0.4313688	-1.5045432
N	0.6339117	-0.2546469	0.0000000
C	1.3199464	-0.2499592	1.1385503
C	1.3199464	-0.2499592	-1.1385503
C	2.7049360	-0.2395308	1.1915103
H	0.7354916	-0.2511934	2.0539325
C	2.7049360	-0.2395308	-1.1915103
H	0.7354916	-0.2511934	-2.0539325
C	3.4116584	-0.2344424	0.0000000
H	3.2123103	-0.2348177	2.1483147
H	3.2123103	-0.2348177	-2.1483147
H	4.4954772	-0.2263015	0.0000000
F	-3.5131477	0.9047864	1.6203170
F	-1.4435801	1.4390915	1.3629305
F	-1.9729549	-0.2251694	2.6248036
F	-1.4435801	1.4390915	-1.3629305
F	-3.5131477	0.9047864	-1.6203170
F	-1.9729549	-0.2251694	-2.6248036

14

Se	-1.0344129	-0.8891802	0.0000000
O	-2.6559712	-0.9550230	0.0000000
C	-0.7053145	0.4837361	1.4951138
C	-0.7053145	0.4837361	-1.4951138
N	1.9772679	-0.9221054	0.0000000
H	2.3303683	-1.8712768	0.0000000
H	2.3595340	-0.4555514	0.8136598
H	2.3595340	-0.4555514	-0.8136598
F	-0.6600559	-0.2087096	2.6322062
F	0.4201276	1.1791033	1.3857609
F	-1.7229172	1.3202143	1.5437145
F	0.4201276	1.1791033	-1.3857609
F	-1.7229172	1.3202143	-1.5437145
F	-0.6600559	-0.2087096	-2.6322062

15

Se	-0.3212097	-1.1428138	0.0000000
O	-1.9655115	-1.1563765	0.0000000
C	-0.0352483	0.2240249	1.5192841
C	-0.0352483	0.2240249	-1.5192841
Cl	2.4717301	-1.1954357	0.0000000
F	0.2541595	-0.4729655	2.6227698
F	0.8874126	1.1527462	1.3561606
F	-1.1988282	0.8435073	1.7308888
F	0.8874126	1.1527462	-1.3561606
F	0.2541595	-0.4729655	-2.6227698
F	-1.1988282	0.8435073	-1.7308888

16

Se	-0.2182708	-1.1675789	0.0000000
O	-1.8475381	-1.3717143	0.0000000
C	-0.0817493	0.2321651	1.5089241
C	-0.0817493	0.2321651	-1.5089241
Br	2.8074493	-1.0380236	0.0000000
F	0.2053017	-0.4337811	2.6307220
F	-1.2869214	0.7822765	1.6638868
F	0.7925488	1.2079979	1.3620714
F	0.2053017	-0.4337811	-2.6307220
F	-1.2869214	0.7822765	-1.6638868
F	0.7925488	1.2079979	-1.3620714

17

Se	-0.4838889	-1.0537633	0.0000000
O	-2.1523501	-0.9250291	0.0000000
C	0.0452358	0.1378984	1.4283556
C	0.0452358	0.1378984	-1.4283556
O	2.6888849	-1.2154337	0.0000000
H	3.1414576	-1.5852512	-0.7621568
H	3.1414576	-1.5852512	0.7621568
Ag	-3.1629091	0.9635463	0.0000000
Cl	-4.0312962	3.0711956	0.0000000
H	-0.2361123	-0.3336305	-2.3683164
H	-0.2361123	-0.3336305	2.3683164
H	-0.5017950	1.0724760	-1.2917705
H	-0.5017950	1.0724760	1.2917705

H	1.1219936	0.2882493	-1.3669729
H	1.1219936	0.2882493	1.3669729

18

Se	-0.9729253	-1.1713050	0.0000000
O	-2.4244369	-2.0071376	0.0000000
C	-1.2056872	0.1068100	1.4318578
H	-2.1620643	0.6083072	1.2744825
H	-0.3696545	0.8039022	1.4055316
H	-1.2173792	-0.4541511	2.3647232
C	-1.2056872	0.1068100	-1.4318578
H	-1.2173792	-0.4541511	-2.3647232
H	-0.3696545	0.8039022	-1.4055316
H	-2.1620643	0.6083072	-1.2744825
Ag	-4.3071698	-0.9951577	0.0000000
Cl	-6.1886034	0.2883783	0.0000000
N	1.8599609	0.3601689	0.0000000
C	3.0078632	0.3248154	0.0000000
C	4.4548139	0.2770731	0.0000000
H	4.8439669	0.7769474	0.8881779
H	4.7921339	-0.7604670	0.0000000
H	4.8439669	0.7769474	-0.8881779

19

Se	-0.9744992	-1.7068585	0.0000000
O	-2.0089183	-3.0261554	0.0000000
C	-1.6898136	-0.6153301	1.4296925
H	-2.7721018	-0.5764530	1.2937593
H	-1.2399178	0.3736487	1.3690222
H	-1.4443947	-1.1069816	2.3695475
C	-1.6898136	-0.6153301	-1.4296925
H	-1.4443947	-1.1069816	-2.3695475
H	-1.2399178	0.3736487	-1.3690222
H	-2.7721018	-0.5764530	-1.2937593
Ag	-4.1364873	-2.8159125	0.0000000
Cl	-6.3676439	-2.3489603	0.0000000
N	1.0741199	0.5880172	0.0000000
C	1.7013015	0.8731330	1.1374005
C	1.7013015	0.8731330	-1.1374005
C	2.9605203	1.4496266	1.1915608
H	1.1726846	0.6279841	2.0547350
C	2.9605203	1.4496266	-1.1915608
H	1.1726846	0.6279841	-2.0547350
C	3.6032219	1.7442127	0.0000000
H	3.4223659	1.6598056	2.1482606
H	3.4223659	1.6598056	-2.1482606
H	4.5889180	2.1947905	0.0000000

20

Se	-0.0379631	-1.0936473	0.0000000
O	-1.4913956	-1.9258432	0.0000000
C	-0.2751374	0.1863217	1.4305755
H	-1.2389055	0.6749492	1.2768615
H	0.5511903	0.8938082	1.3954551
H	-0.2754293	-0.3701432	2.3661667
C	-0.2751374	0.1863217	-1.4305755
H	-0.2754293	-0.3701432	-2.3661667
H	0.5511903	0.8938082	-1.3954551
H	-1.2389055	0.6749492	-1.2768615
Ag	-3.3765622	-0.9179142	0.0000000
Cl	-5.2552776	0.3684321	0.0000000
N	2.8714782	0.2009851	0.0000000
H	3.2510007	-0.7389560	0.0000000
H	3.2576417	0.6685358	0.8122928
H	3.2576417	0.6685358	-0.8122928

21

Se	0.7400505	-1.0359168	0.0000000
O	-0.8351172	-1.7093667	0.0000000
C	0.5401558	0.2428404	1.4395032
H	-0.4266306	0.7284657	1.2945609
H	1.3869375	0.9235312	1.3957394
H	0.5425041	-0.3274479	2.3672660
C	0.5401558	0.2428404	-1.4395032
H	0.5425041	-0.3274479	-2.3672660
H	1.3869375	0.9235312	-1.3957394
H	-0.4266306	0.7284657	-1.2945609

Ag	-2.6817211	-0.7206691	0.0000000
Cl	-4.7447877	0.3082331	0.0000000
Cl	3.4356420	0.0229408	0.0000000

22

Se	0.6670478	-1.0808373	0.0000000
O	-0.9210337	-1.7089437	0.0000000
C	0.5224935	0.2067408	1.4376537
H	-0.4191693	0.7386779	1.2895096
H	1.3992630	0.8498618	1.3969053
H	0.4978121	-0.3616152	2.3661782
C	0.5224935	0.2067408	-1.4376537
H	0.4978121	-0.3616152	-2.3661782
H	1.3992630	0.8498618	-1.3969053
H	-0.4191693	0.7386779	-1.2895096
Ag	-2.7099724	-0.6097825	0.0000000
Cl	-4.6745141	0.5869711	0.0000000
Br	3.6376738	-0.0547381	0.0000000

23

Se	-0.3349093	-1.0712853	0.0000000
O	-1.9734751	-0.9373623	0.0000000
C	0.1698237	0.2307632	1.5043021
C	0.1698237	0.2307632	-1.5043021
O	2.5084760	-1.6932759	0.0000000
H	3.0453338	-1.4630671	-0.7630233
H	3.0453338	-1.4630671	0.7630233
F	-0.0191472	-0.4315768	-2.6351057
F	-0.6182765	1.2811869	1.4733952
F	-0.6182765	1.2811869	-1.4733952
F	1.4316303	0.6184118	-1.4321516
F	1.4316303	0.6184118	1.4321516
F	-0.0191472	-0.4315768	2.6351057
Ag	-3.3881274	0.7330841	0.0000000
Cl	-4.8306924	2.4974031	0.0000000

24

Se	-1.0726555	-1.0907856	0.0000000
O	-2.6564310	-1.5339344	0.0000000
C	-1.0451191	0.2926957	1.5207760
C	-1.0451191	0.2926957	-1.5207760
Ag	-4.5942059	-0.5165756	0.0000000
Cl	-6.6329901	0.5103267	0.0000000
F	-2.2343134	0.8483993	-1.6144819
F	-0.7908713	-0.3928872	-2.6264152
F	-0.1296525	1.2257619	-1.3659142
F	-2.2343134	0.8483993	1.6144819
F	-0.7908713	-0.3928872	2.6264152
F	-0.1296525	1.2257619	1.3659142
N	1.8106039	-0.5389040	0.0000000
C	2.9451854	-0.3679110	0.0000000
C	4.3751341	-0.1460216	0.0000000
H	4.6621031	0.4187748	-0.8882215
H	4.6621031	0.4187748	0.8882215
H	4.9010655	-1.1016834	0.0000000

25

Se	-0.9352801	-1.5632256	0.0000000
O	-2.0625047	-2.7649890	0.0000000
C	-1.6606563	-0.3722365	1.5127683
C	-1.6606563	-0.3722365	-1.5127683
Ag	-4.2323142	-2.9580720	0.0000000
Cl	-6.5013228	-3.1918178	0.0000000
N	1.1552736	0.3144770	0.0000000
C	1.7526531	0.6509914	1.1402167
C	1.7526531	0.6509914	-1.1402167
C	2.9547935	1.3372419	1.1915227
H	1.2470738	0.3637325	2.0568699
C	2.9547935	1.3372419	-1.1915227
H	1.2470738	0.3637325	-2.0568699
C	3.5674143	1.6892598	0.0000000
H	3.3949660	1.5887688	2.1482249
H	3.3949660	1.5887688	-2.1482249
H	4.5072434	2.2289758	0.0000000
F	-2.9608689	-0.5449260	-1.6004706
F	-1.0826896	-0.8107686	-2.6274118
F	-1.3945266	0.9098926	-1.3672325

F	-2.9608689	-0.5449260	1.6004706
F	-1.0826896	-0.8107686	2.6274118
F	-1.3945266	0.9098926	1.3672325

26

Se	-0.1389475	-1.0793133	0.0000000
O	-1.7461593	-1.4391977	0.0000000
C	-0.0598807	0.2941116	1.5329411
C	-0.0598807	0.2941116	-1.5329411
Ag	-3.6261721	-0.3316378	0.0000000
Cl	-5.5673258	0.8639191	0.0000000
F	-1.2582234	0.8034263	-1.7029465
F	0.2861942	-0.3960304	-2.6131218
F	0.8097534	1.2723187	-1.3546577
F	-1.2582234	0.8034263	1.7029465
F	0.2861942	-0.3960304	2.6131218
F	0.8097534	1.2723187	1.3546577
N	2.6025919	-0.5269284	0.0000000
H	3.0895735	-1.4154894	0.0000000
H	2.9153761	-0.0095024	0.8133249
H	2.9153761	-0.0095024	-0.8133249

27

Se	0.6531416	-1.1275461	0.0000000
O	-1.0043717	-1.4273497	0.0000000
C	0.6663131	0.2506255	1.5265520
C	0.6663131	0.2506255	-1.5265520
Ag	-2.9307488	-0.5469837	0.0000000
Cl	-5.1091690	0.2072790	0.0000000
F	-0.5725907	0.7028693	-1.6946597
F	1.0180386	-0.4092192	-2.6281712
F	1.4607249	1.2880173	-1.3735780
F	-0.5725907	0.7028693	1.6946597
F	1.0180386	-0.4092192	2.6281712
F	1.4607249	1.2880173	1.3735780
Cl	3.2461759	-0.7699851	0.0000000

28

Se	0.6605761	-1.1156973	0.0000000
O	-0.9838863	-1.4658865	0.0000000
C	0.6415559	0.2710872	1.5205320
C	0.6415559	0.2710872	-1.5205320
Ag	-2.9286729	-0.6103233	0.0000000
Cl	-5.1133466	0.1212259	0.0000000
F	-0.6025084	0.7175608	-1.6595347
F	0.9750625	-0.3816794	-2.6301164
F	1.4349537	1.3091954	-1.3728459
F	-0.6025084	0.7175608	1.6595347
F	0.9750625	-0.3816794	2.6301164
F	1.4349537	1.3091954	1.3728459
Br	3.4672024	-0.7616467	0.0000000
